# 25-hydroxycholesterol enhances cytokine release and toll-like receptor 3 response in airway epithelial cells

**DOI:** 10.1186/1465-9921-13-63

**Published:** 2012-07-31

**Authors:** Akira Koarai, Satoru Yanagisawa, Hisatoshi Sugiura, Tomohiro Ichikawa, Takashi Kikuchi, Kanako Furukawa, Keiichiro Akamatsu, Tsunahiko Hirano, Masanori Nakanishi, Kazuto Matsunaga, Yoshiaki Minakata, Masakazu Ichinose

**Affiliations:** 1 Third Department of Internal Medicine, Wakayama Medical University, 811-1 Kimiidera, Wakayama, 641-8509, Japan; 2 Department of Respiratory Medicine, Tohoku University Graduate School of Medicine, Sendai, Japan

**Keywords:** Airway inflammation, Interferon regulatory factor 3, Interleukin-8, Nuclear factor-kappa B, Oxysterol

## Abstract

**Background:**

25-hydroxycholesterol (25-HC) is one of the oxysterols, which are oxidized derivatives of cholesterol, and has been reported to be involved in the pathogenesis of atherosclerosis and Alzheimer’s disease. In lung, the possible involvement of 25-HC in airway diseases has been revealed. In the present study, we examined whether 25-HC affects the release of cytokines and also modulates the responses of toll-like receptor 3 (TLR3) in airway epithelial cells.

**Methods:**

The effect of 25-HC on the release of cytokines from primary human bronchial epithelial cells after stimulation with or without polyinosine-polycytidylic acid [poly(I:C)], a ligand for TLR3, and the signal transduction were examined.

**Results:**

25-HC significantly potentiated the release of interleukin-8 (IL-8) and IL-6 from the cells. This effect was more potent compared with that of other oxysterols, 22-HC and 27-HC. GW3965 and TO901317, synthetic agonists of liver X receptors that are receptors for oxysterols, did not augment the IL-8 release. 25-HC enhanced the nuclear factor-kappa B (NF-κB) DNA binding activity and translocation of phosphorylated c-Jun into the nucleus. The release of IL-8 was inhibited by the NF-κB inhibitor, caffeic acid phenethyl ester (CAPE), an inhibitor of nuclear factor kappa-B alpha (IκBα) inhibitor, BAY 11–7085, and an inhibitor of nuclear factor kappa-B kinase-2 (IKK-2) inhibitor, SC-514, but not by a c-Jun N-terminal kinase (JNK) inhibitory peptide, L-JNKi1. 25-HC significantly potentiated IL-8 release in poly(I:C)-treated cells and the augmentation was inhibited by CAPE, BAY 11–7085, and SC-514. Furthermore, 25-HC potentiated the translocation of interferon regulatory factor 3 into the nucleus and the release of interferon-beta (IFN-β) in poly(I:C)-treated cells.

**Conclusions:**

These data demonstrated that 25-HC augments the release of IL-8 and IL-6 via NF-κB signalling pathway and enhances the release of IL-8 and IFN-β after stimulation of TLR3 in airway epithelial cells. 25-HC may be involved in the neutrophilic airway inflammation through the stimulant effect of IL-8 and IL-6 release and also potentiate the TLR3-mediated innate immunity in airway diseases.

## Background

25-hydroxycholesterol (25-HC) is one of the oxysterols, which are oxidized derivatives of cholesterol and are important modulators of cholesterol metabolism [1]. 25-HC is produced from cholesterol by cholesterol 25-hydroxylase, which is detected in several cell types including macrophages [2]. 25-HC has been reported to be mainly involved in the pathogenesis of atherosclerosis [3] and Alzheimer’s disease [4] affecting various aspects such as cytokine release [5] and the imbalance between matrix metalloproteinases and specific tissue inhibitors of metalloproteinases [6] in macrophage lineage cells. These effects have been reported to be mediated by one type of nuclear receptors, liver X receptors [7] or via signalling pathways including nuclear factor-kappa B (NF-κB) [8,9], c-Jun N-terminal kinase (JNK) and mitogenic extracellular kinase/extracellular signal-regulated kinase1/2 (MEK/ERK1/2) [5,10]. In addition, a recent report demonstrated that 25-HC also affects immune systems via the suppression of immunoglobulin A production [2].

In lung, the possible involvement of 25-HC in airway diseases has been revealed. Recently, we demonstrated that the expression of cholesterol 25-hydroxylase in alveolar macrophages and pneumocytes in human lung tissues and the level of 25-HC in sputum from patients with chronic obstructive pulmonary disease (COPD) are increased compared to non-smoker control subjects [11]. In addition, the concentrations of 25-HC in sputum were significantly correlated with the sputum interleukin-8 (IL-8) levels and neutrophil counts [11]. These results suggest that 25-HC might modulate neutrophilic airway inflammation in lung diseases.

Airway epithelial cells are one of the key cells in the pathogenesis of airway diseases through the release of proinflammatory cytokines including IL-1β, IL-6 and neutrophil chemotactic factor, IL-8 after stimulation such as by allergen and oxidant contained in air pollution [12]. In addition, airway epithelial cells are one of the first defenses against inhaled microorganisms via the innate immune systems, including toll-like receptors (TLRs), through recognizing pathogen-associated molecular patterns [13]. Virus infections are a major cause of exacerbations of the airway diseases that are characterized by the accumulation of neutrophils in the airways, and preventing such exacerbations is necessary to inhibit the progression of the disease [14,15]. Recently, TLR3 has been demonstrated to react to double-stranded RNA (dsRNA) and to be involved in the immune responses after viral infections [13]. Activation of TLR3 stimulates the production of inflammatory cytokines including IL-8 and type I interferons (IFNs) via NF-κB and interferon regulatory factor 3 (IRF3) pathway [13] and the enhancement of TLR3 response has been demonstrated in airway diseases [16,17].

In airway diseases, including COPD, it is known that there is overproduction of 25-HC in the airways that is correlated with neutrophilia, and that the airway epithelial cells have an important role in the pathogenesis of the diseases and in the exacerbations. However, the effects of 25-HC on airway epithelial cells and in modulating the TLR3 response, which could be involved in the mechanism of the exacerbation, have not been elucidated. The present study, therefore, was designed to determine the following: 1) whether 25-HC could affect the cytokine release including IL-8 in human airway epithelial cells; 2) which signal pathway is involved in this mechanism; 3) whether 25-HC could affect the TLR3-mediated IL-8 and IFN-β release in human airway epithelial cells using polyinosine-polycytidylic acid [poly(I:C)], a synthetic analog of viral dsRNA and a ligand of TLR3.

## Materials and methods

### Materials

Commercially available reagents were obtained as follows: poly(I:C) was purchased from Amersham Biosciences (Piscataway, NJ); caffeic acid phenethyl ester (CAPE), BAY 11–7085 and SC-514 were purchased from Calbiochem (La Jolla, CA); serum-free Keratinocyte Basal Medium and its supplement including recombinant epidermal growth factor and bovine pituitary extract were purchased from Invitrogen Life Technologies (Grand Island, NY); 22(R)-HC, 25-HC, GW3965, TO901317, SB203580, U0126, HBSS, BSA, phenylmethylsulfonyl fluoride, aprotinin, leupeptin, thiazolyl blue tetrazolium (MTT) and dimethyl sulphoxide (DMSO) were purchased from Sigma Aldrich, Inc. (St. Louis, MO). 27-HC and L-JNKi1 were purchased from Avanti Polar Lipids, Inc.(Alabaster, AL) and Enzo Life Sciences, Inc. (Exeter, UK), respectively.

### *P*reparation of epithelial cells

Four strains of primary human bronchial epithelial cells (HBEpC) from normal subjects were purchased from Cell Aplications Inc. (San Diego, CA) and ScienCell research laboratories (Carlsbad, CA). HBEpC (passages 3–6) were cultured in serum-free Keratinocyte Basal Medium supplemented with 10 ng/ml recombinant epidermal growth factor and 30 μg/ml bovine pituitary extract. Cells were cultured at 37°C in a humidified atmosphere of 5% CO_2_ and passaged. Cells were routinely grown to 80% confluence and growth was arrested overnight prior to the experimental procedures by transfer to growth factor-free media [17]. Cells were cultured in 96-well plates for investigating the effect of 25-HC or other ligand-induced cytokine release. To investigate the effect of 25-HC or other ligand-induced cytokine release, the supernatants were harvested at 24 h after the treatment and stored at −80°C until the measurement. To estimate the effect of 25-HC on the poly(I:C)-induced cytokine release, 25-HC was added to the media 15 min prior to the treatment with poly(I:C). To evaluate the effects of CAPE, BAY 11–7085, SC-514, SB203580, U0126 or L-JNKi1, these drugs were added to the media at various concentrations, 30 min or 60 min (BAY 11–7085 and SC-514) prior to 25-HC or poly(I:C) treatment.

### Cell viability assay

The cell viability was estimated by thiazolyl blue tetrazolium (MTT) assay according to previous study [17].

### Measurement of cytokines

The amounts of IL-8 and IFN-β were measured in the supernatants using Enzyme-Linked Immunosorbent Assay (ELISA) Kit (R&D Systems, Abingdon, UK and Thermo Fisher Scientific Inc., Rockford, IL). The amounts of IL-6 and IL-1β were measured in the supernatants using Cytometric Bead Array kit (BD Biosciences, San Jose, CA) according to the manufacturer’s instructions.

### NF-κB p65 DNA binding activity

To assess NF-κB p65 DNA binding activity, the nuclear fraction was obtained after 0–120 min incubation with poly(I:C) or vehicle and evaluated by an NF-κB p65 transcription factor assay kit, which is based on the method of ELISA (Cayman Chemical Company, Ann Arbor, MI) according to the manufacturer’s instructions [17].

### Detection of pc-Jun and IRF-3 by nuclear extraction and immunoblotting

Cells were treated with 10 μg/ml 25-HC for 0–120 min. After washing with HBSS, cells were homogenized in cell lysis buffer to obtain the nuclear fraction. Samples were separated by electrophoresis and blotted on a PVDF membrane. The following antibodies were used for detection of the target proteins: mouse monoclonal anti-pc-Jun antibody (1:200 dilution; Santa Cruz Biotechnology), rabbit polyclonal anti–IRF-3 antibody (1:1000 dilution; Santa Cruz Biotechnology), or mouse monoclonal anti-lamin A/C antibody (1:750 dilution; Santa Cruz Biotechnology). Appropriate peroxidase-conjugated secondary antibodies were used. Binding antibodies were detected using ECL-plus (Amersham Biosciences, Buckinghamshire, UK) and visualized with a chemiluminescence imaging system (Luminocapture AE6955, Atto Co., Tokyo, Japan). Each band intensity was quantified by densitometry (Image J, NIH, Frederick, MD).

### Detection of TLR3 expression

Cells were treated with 10 μg/ml poly(I:C) in the presence or absence of 10 μM 25-HC for 24 h. After washing with HBSS, cells were homogenized in cell lysis buffer (0.05% Triton X, 35 mM Tris–HCl, pH 7.4, 0.4 mM EGTA, 10 mM MgCl_2_, 1 μM phenylmethylsulfonyl fluoride, 100 μg/ml aprotinin and 1 μg/ml leupeptin) at 4°C. Samples were solubilized in SDS-PAGE sample buffer. Equal amounts of protein were separated by electrophoresis on 15% SDS polyacrylamide gels and blotted on a PVDF membrane. Mouse monoclonal anti-TLR3 antibody (1:250 dilution; Imgenex Corporation, San Diego, CA) or mouse monoclonal anti-β-actin antibody (1:10000 dilution; Sigma, St. Louis, MO) were used for the detection of target proteins. Peroxidase-conjugated appropriate secondary antibodies were used. The following detection and visualization procedures were performed the same as for NF-κB immunoblotting.

### Statistical analysis

Data were expressed as means ± SEM. GraphPad Prism (GraphPad Software Inc., San Diego, CA) was used to perform all statistical tests. Experiments with multiple comparisons were evaluated by one way analysis of variance (ANOVA) followed by Bonferroni's test or Dunnett's test to adjust for multiple comparisons. An unpaired Student's t-test was used for single comparisons. Probability values of less than 0.05 were considered significant.

## Results

### Effect of oxysterols or liver X receptor ligands on IL-8 release in HBEpC

**Figure 1:**
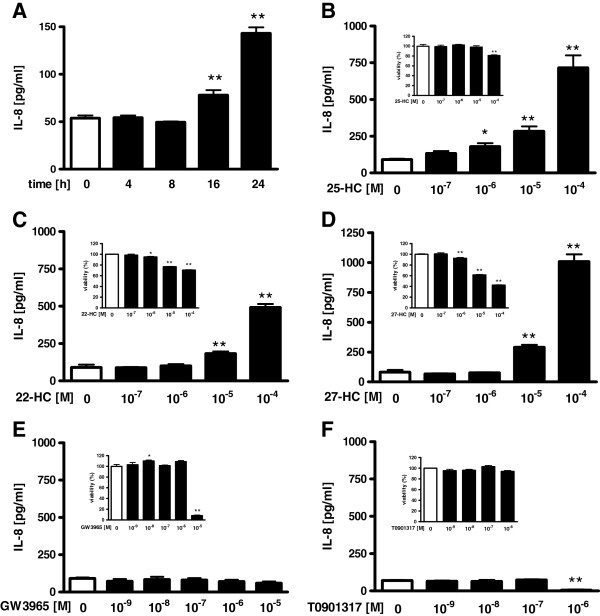
**Effect of oxysterols and liver X receptor ligands on IL-8 release in HBEpC.(A)** Cells were treated with 10^-5^ M of 25-hydroxycholesterol (25-HC). At various time points after the incubation, supernatants were harvested and assayed for IL-8 by ELISA. (B-F) Cells were treated with various concentrations of 25-HC **(B)**, 22-hydroxycholesterol (22-HC) **(C)**, 27-hydroxycholesterol (27-HC) **(D)**, liver X receptor ligands, GW3965 **(E)**, or TO901317 **(F)**. After 24 h, supernatants were harvested and assayed for IL-8. Inserted figures show the effects of hydroxycholesterols and liver X receptor ligands on cell viability. Cells were incubated with various concentrations of 25-HC **(B)**, 22-HC **(C)**, 27-HC **(D)**, GW3965 **(E)**, TO901317 **(F)** or vehicle for 24 h. Cell viability was assessed by MTT assay. Cell viability is calculated as a percentage of the viable cells in the vehicle treated group. The data are expressed as mean values ± SEM for three to four separate experiments with HBEpC from two donors. *p < 0.05, **p < 0.01; compared with the values of control.

To confirm whether 25-HC affects the function of airway epithelial cells, we investigated the effect of 25-HC on the release of IL-8 from HBEpC. 25-HC significantly increased the release of IL-8 from the cells in a time- and concentration-dependent manner (Figure 1A-B). Next, we compared the effect of 25-HC with other oxysterols, 22-HC, which is a potent activator of liver X receptors [18-20], and 27-HC, which is the most abundant oxysterol in the bloodstream [21]. 22-HC and 27-HC also increased the release of IL-8 from the epithelial cells (Figure 1C-D). This augmented effect by 22-HC and 27-HC was not detected at the concentration of 10^-6^ M compared with that of 25-HC, suggesting that the augmented effect of 22-HC and 27-HC on IL-8 release was less potent compared with that of 25-HC. Concerning the effect of oxysterols on the cell viability, the cell viability was decreased by about 20 percent only at 10^-4^ M of 25-HC. On the other hand, both 22-HC and 27-HC decreased the cell viability at lower concentrations from 10^-6^ M to 10^-4^ M than did 25-HC. 

Previously, 25-HC has been reported to react via liver X receptors [7]. To confirm whether the effect of 25-HC on IL-8 release is mediated via liver X receptors, we used agonists of liver X receptors, GW3965 and TO901317. However, neither GW3965 nor TO901317 augmented the release of IL-8 (Figure 1E-F), and TO901317 significantly inhibited the release of IL-8 at 10^-6^ M (Figure 1F).

### Effect of 25-HC on the release of other cytokines in HBEpC

**Figure 2:**
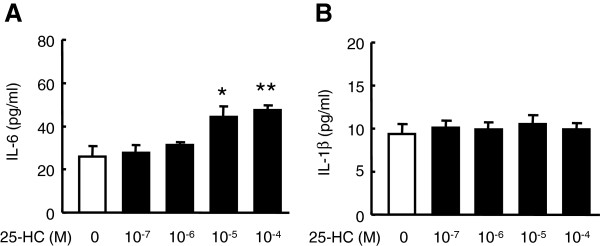
**Effect of 25-HC on other cytokines release in HBEpC.** Cells were treated with various concentrations of 25-HC. After 24 h, supernatants were harvested and assayed for IL-6 **(A)** and IL-1β **(B)** using a Cytometric Bead Array kit. The data are expressed as mean values ± SEM for three to four separate experiments with HBEpC from two donors. *p < 0.05, **p < 0.01, compared with the values of control. BAY = BAY 11–7085.

Furthermore, we examined the effect of 25-HC on the release of IL-6 and IL-1β, which have been reported to be released from airway epithelial cells after various types of stimulation [12,22]. 25-HC significantly augmented the release of IL-6, but not that of IL-1β from HBEpC (Figure 2A-B). 

### Involvement of NF-κB and mitogen-actiated protein kinase (p38, MEK1/2, JNK) signalling pathways in 25-HC-stimulated IL-8 release

**Figure 3:**
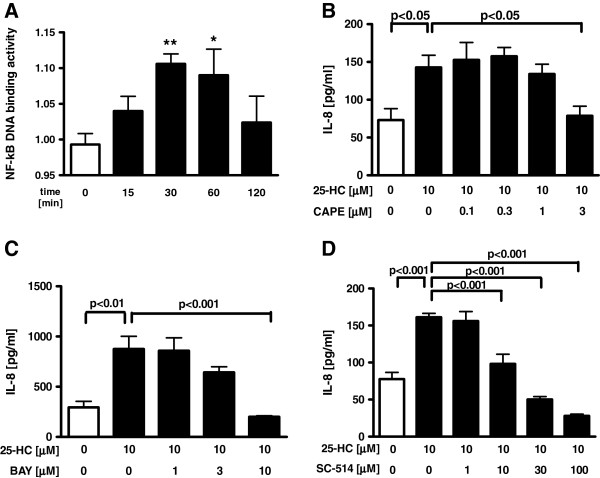
**Involvement of NF-κB signalling pathways in 25-HC-augmented IL-8 release.(A)** Cells were treated with 10 μM 25-HC or vehicle. At various time points, the nuclear fraction of the cell lysates was obtained. NF-κB DNA binding activity was evaluated by ELISA. The data are expressed as mean values ± SEM for three to seven separate experiments with HBEpC from three donors. (B-D) Effect of NF-κB, IκBα and IKK-2 inhibitor on 25-HC-augmented IL-8 release in HBEpC. Cells were treated with 10 μM 25-HC or vehicle in the presence of a NF-κB inhibitor, caffeic acid phenethyl ester (CAPE) **(B)**, an IκBα inhibitor, BAY 11–7085 **(C)** and an IKK-2 inhibitor, SC-514 **(D)**. After 24 h, the supernatants were assayed for IL-8 by ELISA. The data are expressed as mean values ± SEM for three to four separate experiments with HBEpC from two donors. *p < 0.05, **p < 0.01, compared with the values of control at 0 min. NF-κB = nuclear factor-kappa B.

To explore the mechanism of the 25-HC-augmented IL-8 release in the HBEpC, the effects of 25-HC on NF-κB and JNK signalling pathways were evaluated. Treatment with 25-HC caused a small but significant increase in the NF-κB DNA binding activity after 30 min and 60 min (Figure 3A). Furthermore, pretreatment with a NF-κB inhibitor, caffeic acid phenethyl ester (CAPE), an inhibitor of nuclear factor kappa-B alpha (IκBα) inhibitor, BAY 11–7085 and an inhibitor of nuclear factor kappa-B kinase-2 (IKK-2) inhibitor, SC-514 dose-dependently inhibited the 25-HC-augmented IL-8 release in the cells without affecting the cell viability (Figure 3B-D, Additional file 1: Figure S1A-C).

**Figure 4:**
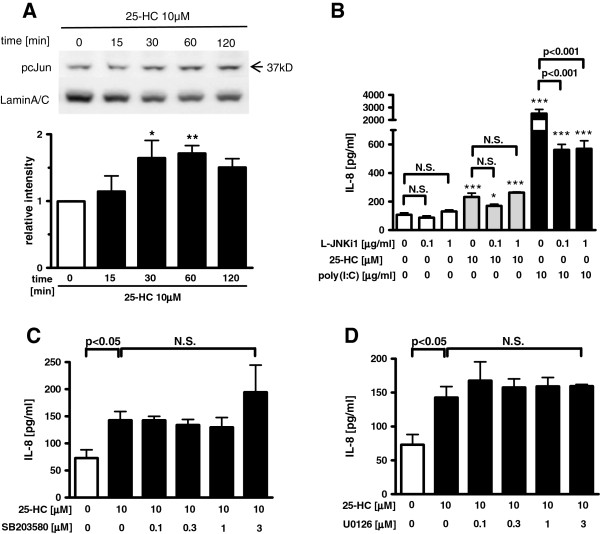
**Involvement of MAPK (p38, MEK1/2, JNK/AP-1) signalling pathways in 25-HC-augmented IL-8 release.(A)** Cells were treated with 10 μM 25-HC or vehicle. At various time points, the nuclear fraction of the cell lysates was obtained. Effect of 25-HC on translocation of phosphorylated c-Jun into the nucleus. Translocation of the phosphorylated c-Jun into the nucleus was evaluated by immunoblotting. Each band intensity was assessed by densitometry. Relative intensity was calculated by dividing the phosphorylated c-Jun band intensity by each appropriate lamin A/C band intensity. The data are expressed as mean values ± SEM for three to seven separate experiments with HBEpC from three donors. **(B-D)** Effect of c-Jun N-terminal kinase (JNK), p38 Mitogen-activated protein kinase (MAPK) or mitogenic extracellular kinase (MEK)1/2 inhibitor on 25-HC-augmented IL-8 release in HBEpC. Cells were treated with 10 μM 25-HC or vehicle in the presence of a JNK inhibitory peptide, L-JNKi1 **(B)**, a p38 MAPK inhibitor, SB203580 **(C)** or a MEK1/2 inhibitor, U0126 **(D)**. After 24 h, the supernatants were assayed for IL-8 by ELISA. *p < 0.05, **p < 0.01, compared with the values of control at 0 min or the values of vehicle-treated cells. +++p < 0.01, compared with the values of 25-HC-treated control cells. The data are expressed as mean values ± SEM for three to four separate experiments with HBEpC from two donors. pcJun = phosphorylated c-Jun. N.S. = not significant.

Concerning the phosphorylated c-Jun, 10 μM 25-HC significantly enhanced phosphorylated c-Jun translocation into the nucleus (Figure 4A). Pre-treatment with a JNK inhibitory peptide, L-JNKi1 significantly inhibited the IL-8 release augmented by the incubation with an agonist of TLR3, poly(I:C) which induces IL-8 release partially via JNK pathway. However, pre-treatment with L-JNKi1 did not inhibit the 25-HC-augmented IL-8 release (Figure 4B).

Furthermore, we examined the involvement of P38 mitogen-actiated protein kinase (MAPK) or MEK/ERK1/2 pathway in this mechanism. However, neither a p38 MAPK inhibitor, SB203580, nor a MEK1/2 inhibitor, U0126, affected the 25-HC-augmented IL-8 release in the cells (Figure 4C-D).

No significant differences in the cell viability were found at these concentrations in the experiments using JNK, MAPK and MEK/ERK1/2 inhibitors (Additional file 1: Figure S1D-F).

### Effect of 25-HC on poly(I:C)-augmented IL-8 release from HBEpC

**Figure 5:**
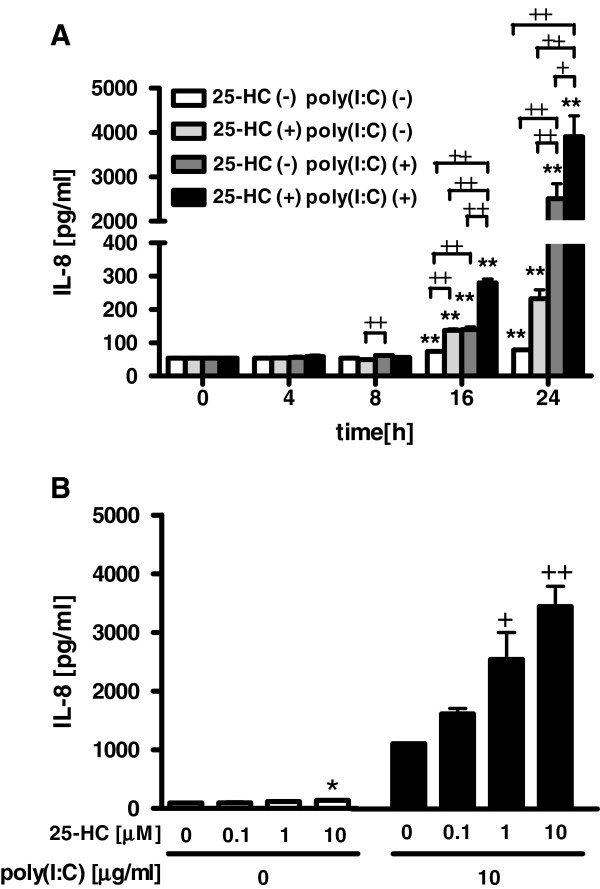
**Effect of 25-HC on poly(I:C)-augmented IL-8 release from HBEpC.(A)** Cells were treated with 10 μM 25-HC or vehicle 15 min prior to the treatment with 10 μg/ml poly(I:C). At various time points after the incubation, supernatants were harvested and assayed for IL-8. **(B)** Cells were treated with various concentrations of 25-HC or vehicle 15 min prior to the treatment with 10 μg/ml poly(I:C). After 24 h, supernatants were harvested and assayed for IL-8. The data are expressed as mean values ± SEM for three to four separate experiments with HBEpC from two donors. *p < 0.05, **p < 0.01, compared with the values of vehicle-treated cells. +p < 0.05, ++p < 0.01, compared with the values of poly(I:C) or 25-HC-treated control cells.

To examine the effect of 25-HC on the response of TLR3 in airway epithelial cells, we investigated the effect of 25-HC on the release of IL-8 from the poly(I:C)-treated HBEpC. 25-HC significantly increased the release of IL-8 from the 10 μg/ml poly(I:C)-treated cells in a time- and concentration-dependent manner (Figure 5A-B).

### Effect of NF-kB on 25-HC-augmented IL-8 release in poly(I:C)-treated cells

**Figure 6:**
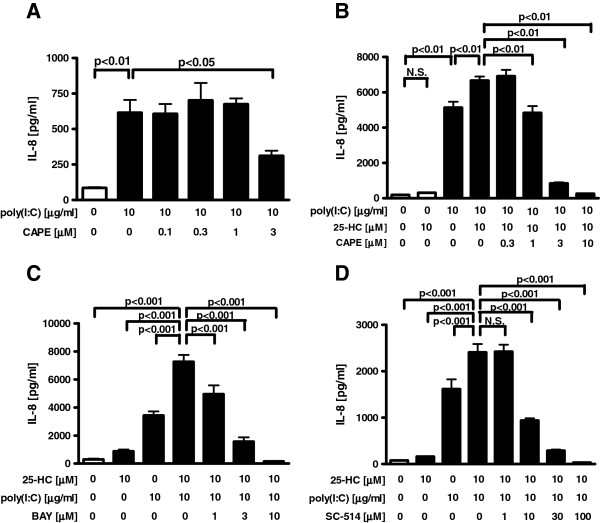
**Effect of NF-κB inhibitors on 25-HC-augmented IL-8 release in poly(I:C)-treated cells.(A)** Effect of a NF-κB inhibitor, CAPE on poly(I:C)-augmented IL-8 release in HBEpC. Cells were treated with various concentrations of CAPE 30 min prior to the treatment with 10 μg/ml poly(I:C). After 24 h, supernatants were harvested and assayed for IL-8. **(B-D)** Effect of NF-κB inhibitors on 25-HC-augmented IL-8 release in poly(I:C)-treated cells. Various concentrations of a NF-κB inhibitor, CAPE **(B)**, an IκBα inhibitor, BAY 11–7085 **(C)** or an IKK-2 inhibitor, SC-514 **(D)** were added before 10 μM 25-HC treatment, and the cells were then cultured in the presence of 10 μg/ml poly(I:C). After 24 h, supernatants were harvested and assayed for IL-8. The data are expressed as mean values ± SEM for three to four separate experiments with HBEpC from two donors. BAY = BAY 11–7085. N.S. = not significant.

NF-κB pathway has been also reported to be involved in the signal pathway after the stimulation of TLR3 [13], and this was confirmed using a NF-κB inhibitor, CAPE. Pretreatment with CAPE significantly decreased the IL-8 release in poly(I:C)-treated cells (Figure 6A). Pretreatment with CAPE slightly but significantly decreased the cell viability in poly(I:C)-treated cells at 0.1 μM , however this effect was small and the decreased cell viability was 78.3% (Additional file 1: Figure S2A). To elucidate the involvement of the NF-κB pathway in the mechanism of the 25-HC-augmented IL-8 release in the TLR3 stimulated cells, we examine the effect of NF-κB inhibitors. Pretreatment with a NF-κB inhibitor, CAPE, an IκBα inhibitor, BAY 11–7085 or an IKK-2 inhibitor, SC-514 significantly inhibited the 25-HC-augmented IL-8 release in poly(I:C)-treated cells without affecting the cell viability (Figure 6B-D, Additional file 1: Figure S2B-D). 

### Effect of 25-HC on interferon regulatory factor 3 (IRF3) and poly(I:C)-induced IFN-β release from HBEpC

**Figure 7:**
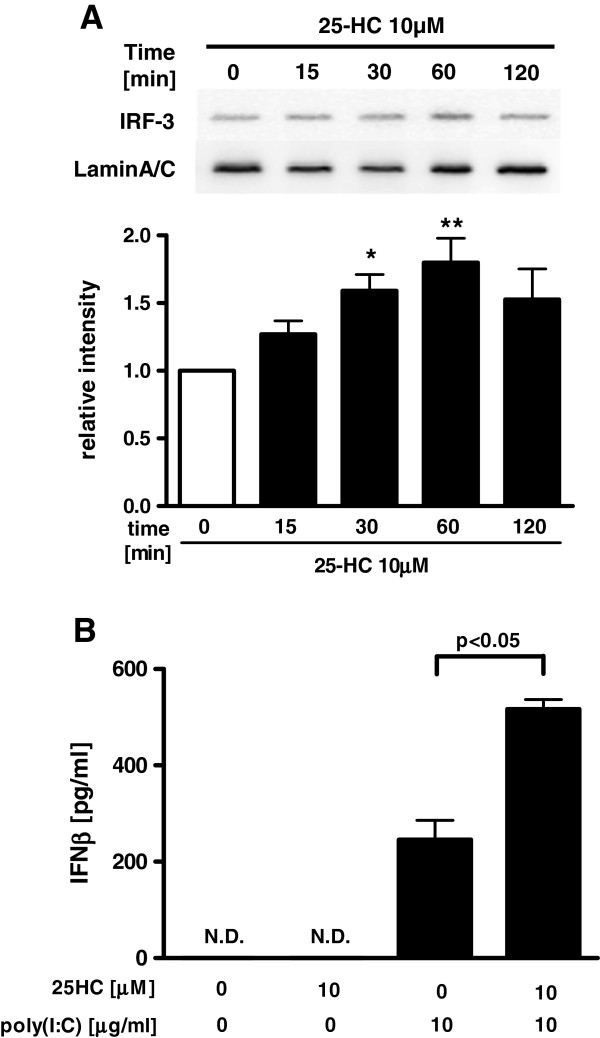
**Effect of 25-HC on Interferon regulatory factor 3 (IRF3) and poly(I:C)-induced IFN-β release from HBEpC.(A)** Effect of 25-HC on translocation of IRF3 into the nucleus. Cells were treated with 10 μM 25-HC or vehicle. At various time points, the nuclear fraction of the cell lysates was obtained. Translocation of IRF3 into the nucleus was evaluated by immunoblotting. Each band intensity was assessed by densitometry. Relative intensity was calculated by dividing the IRF3 band intensity by each appropriate lamin A/C band intensity. **(B)** Effect of 25-HC on poly(I:C)-augmented IFN-β release in HBEpC. Cells were treated with 10 μM 25-HC or vehicle in the presence of 10 μg/ml poly(I:C). After 24 h, the supernatants were assayed for IFN-β by ELISA. *p < 0.05, **p < 0.01, compared with the values of control at 0 min. The data are expressed as mean values ± SEM for four separate experiments with HBEpC from two donors. N.D. = not detectable.

To explore the effect of 25-HC on IRF3 signalling, which is another crucial TLR3 signal pathway that induces the release of IFNs, we investigated the effect of 25-HC on the translocation of IRF-3 into the nucleus and the release of IFN-β from poly(I:C)-treated HBEpC. 25-HC significantly potentiated the translocation of IRF3 into the nucleus after 30 min and 60 min (Figure 7A). 25-HC alone did not affect the release of IFN-β, but pre-treatment with 25-HC significantly increased the release of IFN-β from the 10 μg/ml poly(I:C)-treated cells (Figure 7B).

### Effect of 25-HC on the expression of TLR3 in poly(I:C)-treated HBEpC

**Figure 8:**
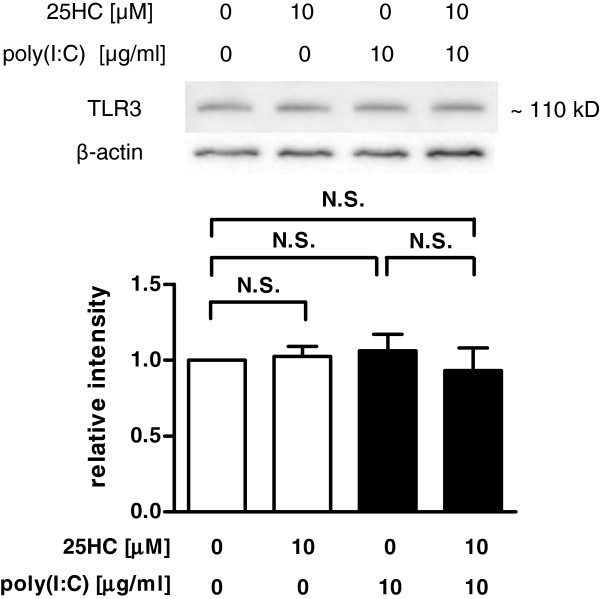
**Effect of 25-HC on TLR3 expression in poly(I:C)-treated primary HBEpC.** Cells were treated with 10 μM 25-HC or vehicle in the presence of 10 μg/ml poly(I:C). After 24 h, the whole cell lysates were obtained. The expression of TLR3 was evaluated by immunoblotting. Each band intensity was assessed by densitometry. Relative intensity was calculated by dividing the TLR3 band intensity by each appropriate β-actin band intensity. The data are expressed as mean values ± SEM for four separate experiments with HBEpC from two donors. **p < 0.01, compared with the values of vehicle-treated cells. +p < 0.05, compared with the values of each group. N.S. = not significant.

To examine the mechanism of the 25-HC-potentiated IL-8 and IFN-β release in the poly(I:C)-treated cells, the effect of 25-HC on the expression of TLR3 in the epithelial cells was evaluated. However, treatment with 10 μM 25-HC or 10 μg/ml poly(I:C) alone or in combination did not affect the expression of TLR3 (Figure 8).

## Discussion

**Figure 9:**
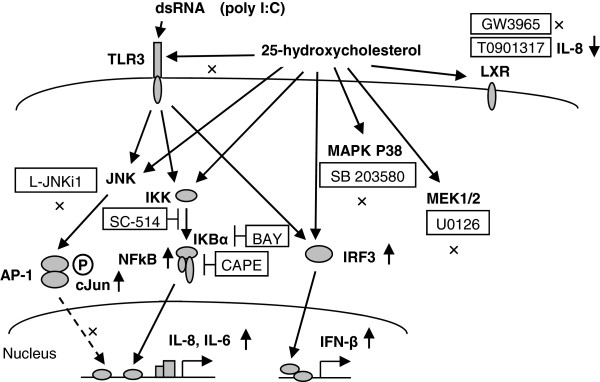
**Schematic representation of the effect of 25-HC on cytokine release and toll-like receptor 3 response.** 25-HC potentiated the release of IL-8 and IL-6 from HBEpC. 25-HC enhanced the NF-κB DNA binding activity and translocation of phosphorylated c-Jun into the nucleus. A NF-κB inhibitor, CAPE, an IκBα inhibitor, BAY 11–7085, an IKK-2 inhibitor and SC-514, except a JNK inhibitory peptide, L-JNKi1 inhibited the release of IL-8. Neither a P38 MAPK inhibitor, SB203580 nor a MEK1/2 inhibitor, U0126, affected the 25-HC-augmented IL-8 release. Agonists of liver X receptors, GW3965 and TO901317, did not enhance IL-8 release, and TO901317 conversely inhibited IL-8 release. Concerning the effect of 25-HC on the TLR3 response, 25-HC potentiated the release of IL-8 stimulated by TLR3 ligand, poly(I:C), and the potentiation was inhibited by CAPE, BAY 11–7085 and SC-514. 25-HC also potentiated the translocation of IRF3 into the nucleus and augmented the release of INF-β stimulated by poly(I:C). However, treatment with 25-HC did not affect the expression of TLR3. These data suggest that 25-HC stimulates the release of IL-8 and IL-6 mainly via NF-κB and enhances the release of IL-8 and INF-β after stimulation of TLR3 in human airway epithelial cells. LXR = Liver X receptor. BAY = BAY 11–8065.

In the present study, we demonstrated for the first time that 25-HC stimulated the release of IL-8 and IL-6 from primary human bronchial epithelial cells, mainly via NF-kB. Furthermore, 25-HC potentiated the release of IL-8 and IFN-β in TLR3 ligand, poly(I:C)-treated cells. These results suggest that 25-HC may be involved in the neutrophilic airway inflammation via IL-8 and IL-6 release from airway epithelial cells and also enhance the TLR3-mediated response in airway epithelial cells (Figure 9).

In the present study, we demonstrated that 25-HC increased IL-8 and IL-6 release from airway epithelial cells, which was supported by previous reports on the effect of 25-HC on IL-8 release in a human macrophage lineage [5] and colon carcinoma cell line [23]. This result may explain why the overproduction of 25-HC was correlated with the neutrophilic airway inflammation in COPD in our previous report [11]. The effective concentration of 25-HC in airway epithelial cells was higher than 10^-6^ M, which was more than 100 times higher than that in the sputum from COPD patients. However, the concentration of 25-HC in sputum might be diluted compared with that released in airways. Therefore, the concentrations of 25-HC we used might potentiate cytokines release from airway epithelial cells in COPD patients. The augmenting effect of 25-HC on IL-8 release was more potent compared to that of the other oxysterols, 22-HC and 27-HC. This result is consistent with those of previous reports on monocyte-derived macrophages and porcine retinal pigment epithelial cells [24,25]. In the present study, the inducing effect of 25-HC on IL-8 release was much weaker than that of poly(I:C), suggesting that 25-HC might be an immune modulator rather than an immune stimulator in airway epithelial cells. However, the amount of IL-8 release in 25-HC-treated airway epithelial cells was not largely different from that in monocytes [10], suggesting that 25-HC might cause the same degree of cellular response in airway epithelial cells as in monocytes/macrophages, which have been thought to be the major target cells of oxysterols. Concerning the effect on cytotoxicity, 25-HC was less cytotoxic compared with 22-HC and 27-HC in airway epithelial cells. In the present study, the decrease of cell viability induced by the treatment with oxysterols was likely associated with the increased IL-8 release. Previous reports demonstrated that oxysterols, including 25-HC, induced IL-8 secretion in parallel with the induction of cell death in monocyte/macrophages [10,24], but the relationship between oxysterols-induced IL-8 secretion and cell death remains unclear. Further studies are needed to clarify this point.

Oxysterols, including 25-HC, have been reported to be ligands of liver X receptors, which regulate cholesterol homeostasis [26]. In the present study, synthetic liver X receptor agonists, GW3965 and TO901317 did not augment IL-8 release and the treatment with TO901317 at 10^-6^ M inhibited the basal release of IL-8, which is inconsistent with the augmenting effect of oxysterols on IL-8 release in airway epithelial cells. However, this discrepancy may be explained by a previous report, which demonstrated that oxysterols induce the expression of inflammatory markers through liver X receptor-independent mechanisms [18]. In the present study, we also demonstrated the augmenting effect of 25-HC on the poly(I:C)-induced IL-8 release in airway epithelial cells. However, several reports have demonstrated the inhibitory effect of oxysterols on the LPS-stimulated IL-1-like activity and inflammatory response in murine macrophages via liver X receptors [27,28]. This discrepancy is probably due to the different design of the experiments. In the present study, the duration of the pre-treatment with oxsterols was only 15 min before TLR3 ligand stimulation, but that of the previous studies that demonstrated an inhibitory effect was more than 1 h; in most studies, 18–22 h incubation was followed by LPS stimulation. However, it remains unclear whether the stimulation of 25-HC in the release of IL-8 is via any receptors except liver X receptors, or indirect effects such as producing oxidative stress by 25-HC [5,9,18,25]. Further studies are needed to clarify these mechanisms.

Although the signaling pathways of 25-HC-augmented IL-8 release remain poorly understood in airway epithelial cells, it could be expected that MAPK (JNK, ERK and p38) signal pathway is involved in the mechanism, because it has been reported that 25-HC enhanced MAPK (JNK, ERK and p38) phosphorylation [5,9,29] and ERK inhibitor inhibited 7β-HC-induced IL-8 release [10] in a human monocyte/macrophage lineage. In airway epithelial cells, these MAPKs are also activated by various stimulations, leading to IL-8 release via a different stimulus-dependent MAPK signaling [30,31]. However, in our present study, the 25-HC-induced IL-8 release in airway epithelial cells was not reduced by treatment with a p38 MAPK inhibitor, SB203580, an ERK inhibitor, U0126, or a JNK inhibitory peptide, L-JNKi1. As described above, we found a distinct difference between monocytes/macrophages and airway epithelial cells in their requirements for MAPK in the 25-HC–mediated signaling pathway. This may reflect cell type- or stimulus-dependent differences in the role of MAPK in IL-8 release. The mechanistic differences between these cells in the 25-HC-mediated signaling pathways should be further elucidated.

In the present study, we demonstrated the involvement of the signalling pathways of NF-κB in the 25-HC-induced IL-8 release in human bronchial epithelial cells. This result is consistent with a previous report which demonstrated that a NF-κB inhibitor diminished oxysterol-induced cytokine stimulation in THP-1 cells [9].

The augmented response of TLR3 in airway epithelial cells could be involved in the exacerbation of airway disease including COPD [16,17,32]. We demonstrated for the first time that 25-HC potentiates TLR3-mediated IL-8 and IFN-β release in airway epithelial cells. The mechanism of the 25-HC-augmented TLR3-mediated IL-8 and IFN-β release might be due to the potentiation of NF-κB and IRF-3, which was suggested by the results that 25-HC enhanced the NF-κB DNA binding activity and the translocation of IRF-3 into nucleus. In addition, there remains a possibility that 25-HC stimulates TLR3 or the upper signal molecule including a Toll/IL-1 receptor domain-containing adapter inducing IFNs (TRIF) for NF-κB and IRF3 signalling pathways. However, 25-HC did not affect the expression of TLR3. As another possibility, melanoma differentiation-associated protein 5 and retinoic acid-inducible gene 1 (RIG-I), which are cytosolic RIG-I-like receptors that can also recognize dsRNA including poly(I:C) [13], might be affected by 25-HC. More research will be needed to precisely define these mechanisms.

## Conclusions

We demonstrated that 25-HC stimulated the release of IL-8 and IL-6, mainly via NF-κB pathway, and enhanced the release of IL-8 and IFN-β after stimulation of TLR3 in human airway epithelial cells. These results suggest that 25-HC may be involved in the neutrophilic airway inflammation through the stimulating effect of IL-8 and IL-6 release, mainly via NF-κB pathway, and also potentiate the TLR3-mediated response in airway epithelial cells.

## Abbreviations

25-HC, 25-hydroxycholesterol; ANOVA, Analysis of variance; COPD, Chronic obstructive pulmonary disease; dsRNA, Double-stranded RNA; ELISA, Enzyme-Linked Immunosorbent Assay; HBEpC, Human bronchial epithelial cells; IκBα, Inhibitor of nuclear factor kappa-B alpha; IKK-2, Inhibitor of nuclear factor kappa-B kinase-2; IL-8, interleukin-8; IRF3, Interferon regulatory factor 3; JNK, c-Jun N-terminal kinase; MAPK, Mitogen-actiated protein kinase; MEK/ERK1/2, Mitogenic extracellular kinase/extracellular signal-regulated kinase1/2; NF-κB, Nuclear factor-kappa B; poly(I:C), Polyinosine-polycytidylic acid; RIG-I, Retinoic acid-inducible gene 1; TLR, Toll-like receptor.

## Competing interests

The authors declare that there are no conflicts of interest to disclose.

## Author’s contributions

AK and SY carried out the data analysis and drafted the manuscript. AK, HS, and MI participated in the conception and design of the original study, and contributed substantially to the manuscript. TI, TK, KF, KA, TH, MN, KM, and YM assisted with data analysis and interpretation, and supervised statistical analysis. All authors have given final approval of the version to be published.

## Supplementary Material

Additional file 1 **Figure S1.** Effect of NF-κB and MAPK inhibitors on cell viability in 25-HC-treated HBEpC. Cells were treated with 10 μM 25-HC or vehicle in the presence of an NF-kB inhibitor, caffeic acid phenethyl ester (CAPE)(A), an IκBα inhibitor, BAY 11-7085 (B), an IKK-2 inhibitor, SC-514 (C), a JNK inhibitory peptide, L-JNKi1 (D), a p38 MAPK inhibitor, SB203580 (E) or a MEK1/2 inhibitor, U0126 (F). After 24 h, the cell viability was assessed by MTT assay. Cell viability is calculated as a percentage of the viable cells in the vehicle treated group. The data are expressed as mean values ± SEM for four separate experiments with HBEpC from two donors. BAY = BAY 11-7085. N.S. = not significant. Figure S2. Effect of NF-κB inhibitors on cell viability in 25-HC and poly(I:C)-treated cells. (A) Effect of an NF-κB inhibitor, CAPE on cell viability in poly(I:C)-treated HBEpC. Cells were treated with various concentrations of CAPE 30 min prior to the treatment with 10 μg/ml poly(I:C). After 24 h, cell viability was assessed by MTT assay. (B-D) Effect of the NF-kB inhibitor on cell viability in 25-HC and poly(I:C)-treated cells. Various concentrations of the NF-κB inhibitor, CAPE (B), an IκBα inhibitor, BAY 11-7085 (C) or an IKK-2 inhibitor, SC-514 (D) were added before 10 μM 25-HC treatment, and the cells were then cultured in the presence of 10 μg/ml poly(I:C). After 24 h, cell viability was assessed by MTT assay. Cell viability is calculated as a percentage of the viable cells in the vehicle treated group. The data are expressed as mean values ± SEM for three to four separate experiments with HBEpC from two donors. BAY = BAY 11-7085. N.S. = not significant. Click here for file
