# Supplementary material for: 25-hydroxycholesterol enhances cytokine release and toll-like receptor 3 response in airway epithelial cells
Source: Respir Res. 2012 Jul 31;13(1):63. doi: 10.1186/1465-9921-13-63 (PMC3460764; doi:10.1186/1465-9921-13-63)
Supplement: Additional file 1 — Figure S1. Effect of NF-κB and MAPK inhibitors on cell viability in 25-HC-treated HBEpC. Cells were treated with 10 μM 25-HC or vehicle in the presence of an NF-kB inhibitor, caffeic acid phenethyl ester (CAPE)(A), an IκBα inhibitor, BAY 11-7085 (B), an IKK-2 inhibitor, SC-514 (C), a JNK inhibitory peptide, L-JNKi1 (D), a p38 MAPK inhibitor, SB203580 (E) or a MEK1/2 inhibitor, U0126 (F). After 24 h, the cell viability was assessed by MTT assay. Cell viability is calculated as a percentage of the viable cells in the vehicle treated group. The data are expressed as mean values ± SEM for four separate experiments with HBEpC from two donors. BAY = BAY 11-7085. N.S. = not significant. Figure S2. Effect of NF-κB inhibitors on cell viability in 25-HC and poly(I:C)-treated cells. (A) Effect of an NF-κB inhibitor, CAPE on cell viability in poly(I:C)-treated HBEpC. Cells were treated with various concentrations of CAPE 30 min prior to the treatment with 10 μg/ml poly(I:C). After 24 h, cell viability was assessed by MTT assay. (B-D) Effect of the NF-kB inhibitor on cell viability in 25-HC and poly(I:C)-treated cells. Various concentrations of the NF-κB inhibitor, CAPE (B), an IκBα inhibitor, BAY 11-7085 (C) or an IKK-2 inhibitor, SC-514 (D) were added before 10 μM 25-HC treatment, and the cells were then cultured in the presence of 10 μg/ml poly(I:C). After 24 h, cell viability was assessed by MTT assay. Cell viability is calculated as a percentage of the viable cells in the vehicle treated group. The data are expressed as mean values ± SEM for three to four separate experiments with HBEpC from two donors. BAY = BAY 11-7085. N.S. = not significant. [file 1465-9921-13-63-S1.pdf]

25-hydroxycholesterol enhances cytokine release and toll-like receptor 3 response in airway epithelial cells

Akira Koarai[1], Satoru Yanagisawa[1], Hisatoshi Sugiura[1], Tomohiro Ichikawa[1], Takashi Kikuchi[1], Kanako Furukawa[1], Keiichiro Akamatsu[1], Tsunahiko Hirano[1], Masanori Nakanishi[1], Kazuto Matsunaga[1], Yoshiaki Minakata[1] and Masakazu Ichinose[2].

1 Third Department of Internal Medicine, Wakayama Medical University, Wakayama, Japan.

2 Department of Respiratory Medicine, Tohoku University Graduate School of Medicine, Sendai, Japan

Additional file 1

Figure legends

Additional Figure 1. Effect of NF-kB and MAPK inhibitors on cell viability in 25-HC-treated HBEpC. Cells were treated with 10 μM 25-HC or vehicle in the presence of an NF-kB inhibitor, caffeic acid phenethyl ester (CAPE)(A), an IkBα inhibitor, BAY 11-7085 (B), an IKK-2 inhibitor, SC-514 (C), a JNK inhibitory peptide, L-JNKi1 (D), a p38 MAPK inhibitor, SB203580 (E) or a MEK1/2 inhibitor, U0126 (F). After 24 h, the cell viability was assessed by MTT assay. Cell viability is calculated as a percentage of the viable cells in the vehicle treated group. The data are expressed as mean values ± SEM for four separate experiments with HBEpC from two donors. BAY = BAY 11-7085. N.S. = not significant.

Additional Figure 2. Effect of NF-kB inhibitors on cell viability in 25-HC and poly(I:C)-treated cells. (A) Effect of an NF-kB inhibitor, CAPE on cell viability in poly(I:C)-treated HBEpC. Cells were treated with various concentrations of CAPE

30 min prior to the treatment with 10 μg/ml poly(I:C). After 24 h, cell viability was assessed by MTT assay. (B-D) Effect of the NF-kB inhibitor on cell viability in 25-HC and poly(I:C)-treated cells. Various concentrations of the NF-kB inhibitor, CAPE (B), an IkBα inhibitor, BAY 11-7085 (C) or an IKK-2 inhibitor, SC-514 (D) were added before 10 μM 25-HC treatment, and the cells were then cultured in the presence of 10 μg/ml poly(I:C). After 24 h, cell viability was assessed by MTT assay. Cell viability is calculated as a percentage of the viable cells in the vehicle treated group. The data are expressed as mean values ± SEM for three to four separate experiments with HBEpC from two donors. BAY = BAY 11-7085. N.S. = not significant.
